# Supplementary material for: Chronic degenerative failure of salivary glands can be reversed through restoring mitochondrial function
Source: bioRxiv. 2025 Jan 2:2024.12.31.630834. Preprint. [Version 1] doi: 10.1101/2024.12.31.630834 (PMC11722244; doi:10.1101/2024.12.31.630834)
Supplement: Supplement 1 [file NIHPP2024.12.31.630834v1-supplement-1.pdf]

bioRxiv preprint doi: https://doi.org/10.1101/2024.12.31.630834; this version posted January 2, 2025. The copyright holder for this preprint (which was not certified by peer review) is the author/funder, who has granted bioRxiv a license to display the preprint in perpetuity. It is made available under aCC-BY 4.0 International license.

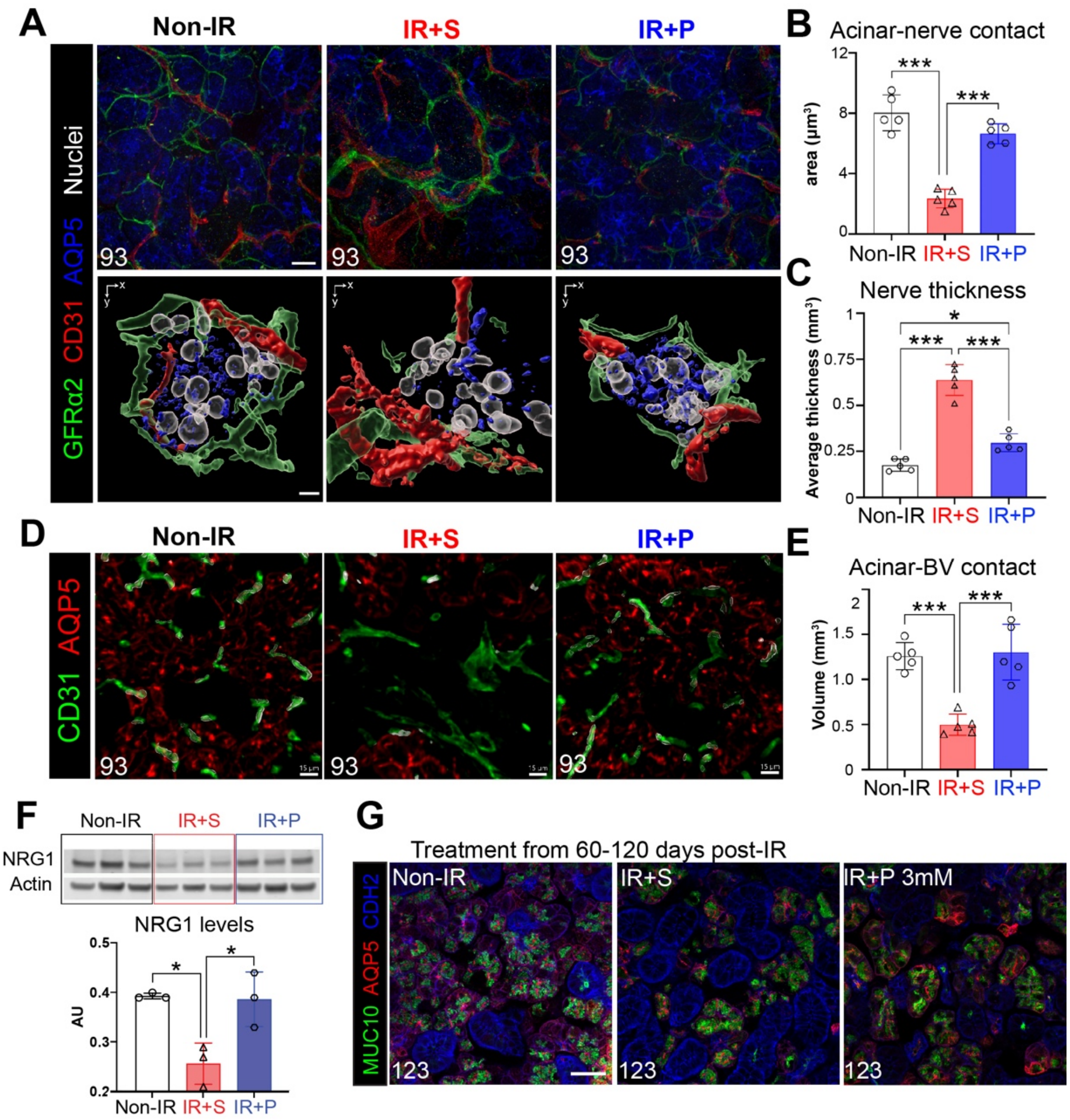

Figure S1. Chronic degeneration is reversed by muscarinic agonism, related to Figure 1. A-C. Immunofluorescent imaging (upper panel) and 3D reconstruction (IMARIS, lower panel) of the acinar (AQP5)-nerve (GFRα2)-capillary (CD31) interactions in SGs of different treatment groups at 93 days post-IR (treatment at 30-90 days post-IR). Quantification of acinar-nerve interactions (B) and large nerve fibers (E). **D-E.** Immunofluorescent imaging (D) and quantification (E) of blood vessels within the SGs of the different experimental groups at 93 days post-IR (treatment at 30-90 days post-IR). **F.** Protein expression and quantification of NRG1 and actin in SG samples at 93 days post-IR. AU, arbitrary units, normalized to actin. **G.** Images of $MUC10^{+}AQP5^{+}$ acinar cells of SGs treated 60-120 days post-IR with 3 mM pilocarpine (IR+ P, 3 mM). Tissue was analyzed at 123 days post-IR. Scale bar in A upper panel and G are 20 µm, A lower panel is 5 µm and D is 15 µm. Mean±SD. *, P<0.05. ***, P<0.001.

bioRxiv preprint doi: https://doi.org/10.1101/2024.12.31.630834; this version posted January 2, 2025. The copyright holder for this preprint (which was not certified by peer review) is the author/funder, who has granted bioRxiv a license to display the preprint in perpetuity. It is made available under aCC-BY 4.0 International license.

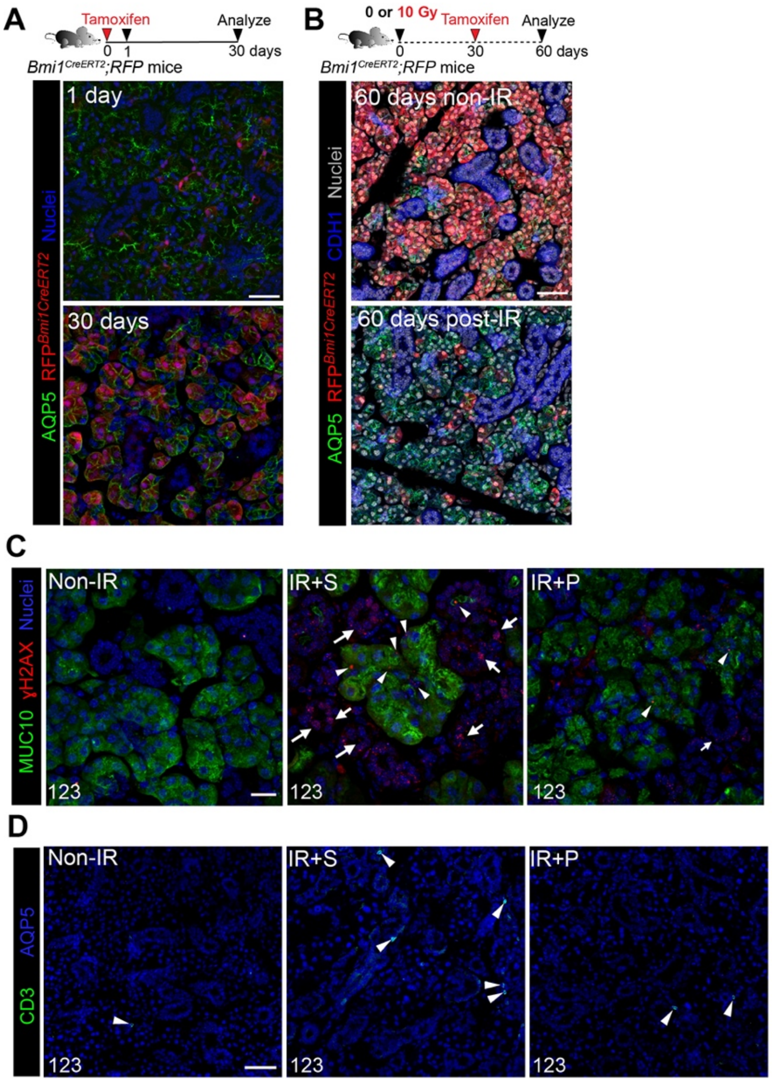

Figure S2. Reversal of chronic SG degeneration is sustained after treatment termination, related to Figure 2. A. Lineage tracing of *Bmi1*^{CreERT2};*Rosa26*^{RFP} mice for 1 and 30 days after Cre activation. Mice were injected with tamoxifen at day 0 and immunostained for AQP5 at day 30. **B.** *Bmi1*^{CreERT2};*Rosa26*^{RFP} mice were treated with 0 or 10 Gy, injected with tamoxifen at 30 days post-IR and immunostained for AQP5 and CDH1 at day 60. **C.** Immunostaining of DNA damage (γH2AX, C) in non-IR, IR+S and IR+P (3 mM, 30-90 days post-IR) at 123-days post-IR (30 days after treatment termination). MUC10 labels acinar cells. Arrowheads mark γH2AX^{+} acinar cells. Arrows mark γH2AX^{+} ducts. **D.** Immunostaining for CD3^{+} T cells in the 3 groups at 123 days post-IR. Arrowheads mark CD3+ cells. Scale bars, 20 μm (A-B), 15 μm (C) and 50 μm (D).

bioRxiv preprint doi: https://doi.org/10.1101/2024.12.31.630834; this version posted January 2, 2025. The copyright holder for this preprint (which was not certified by peer review) is the author/funder, who has granted bioRxiv a license to display the preprint in perpetuity. It is made available under aCC-BY 4.0 International license.

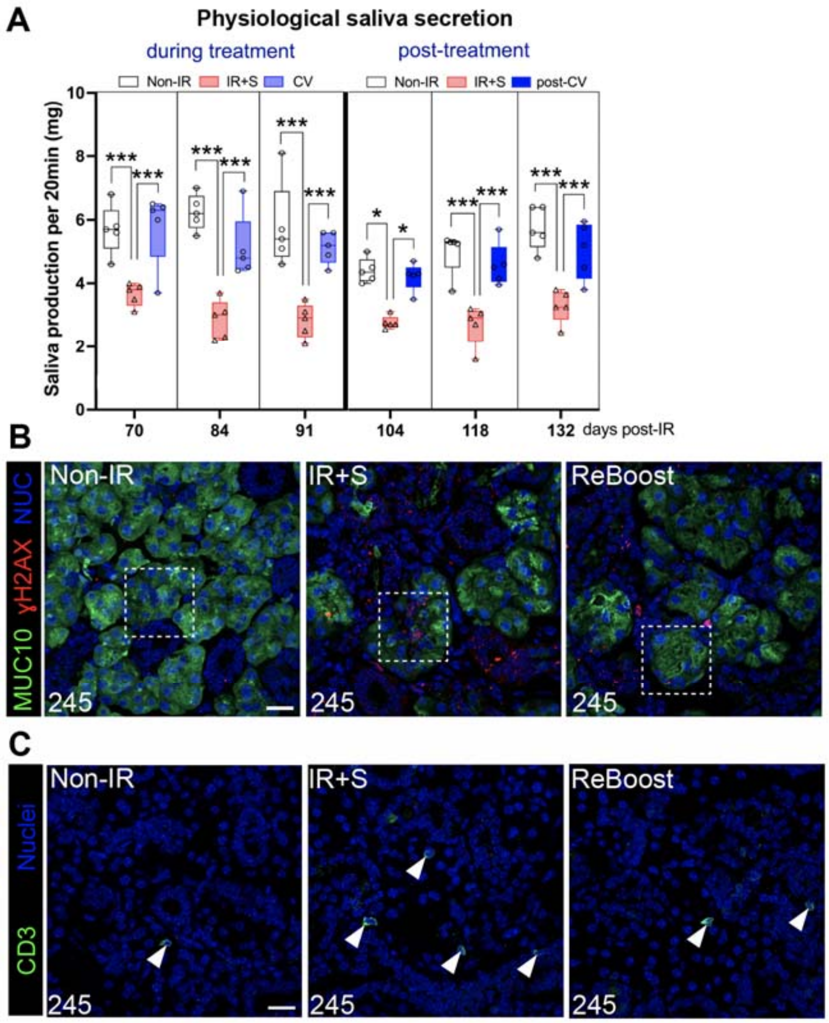

Figure S3. IR SGs remain highly responsive to an additional round of muscarinic treatment delivered months after radiation exposure, related to Figure 3. A. Physiological saliva secretion was measured during treatment (left) and after treatment termination at 90 days post-IR (right). **B.** Detection of DNA damage (γH2AX) foci in acinar cells across the 3 treatment groups (non-IR, IR+S, and IR+ReBoost with CV). Inset shown in Figure 5H. **C.** Immunostaining of CD3+ T cells (arrowheads) in SGs across the groups. Mean±SD. *, P<0.05. **, P<0.01. ***, P<0.001. Scale bars are 20μm.

bioRxiv preprint doi: https://doi.org/10.1101/2024.12.31.630834; this version posted January 2, 2025. The copyright holder for this preprint (which was not certified by peer review) is the author/funder, who has granted bioRxiv a license to display the preprint in perpetuity. It is made available under aCC-BY 4.0 International license.

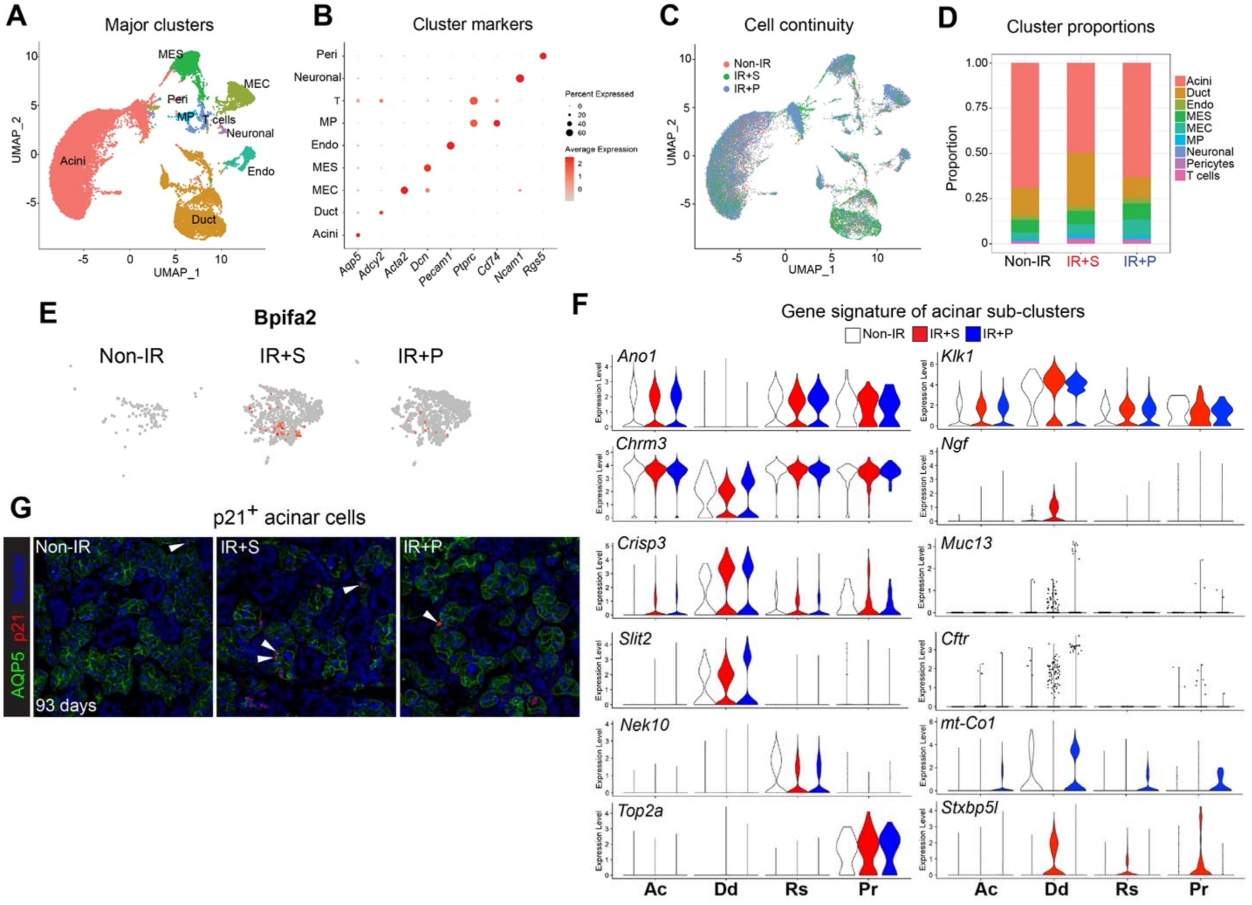

Figure S4. De-differentiated acinar cells are greatly expanded in chronically degenerating SGs, an outcome reversed by muscarinic agonism, related to Figure 4. A. Unbiased cluster analysis**.** MEC, myoepithelial cell. Peri, pericytes. MES, mesenchymal cell. Endo, endothelial. MP, macrophage. T, T cell. **B.** Integrated dot plot presents a specific marker gene for each of the 9 major clusters identified. **C.** UMAP plot of the integrated single nuclei from the 3 treatment groups, non-IR, IR+Saline (S), and IR+Pilocarpine (P, 3 mM) at 93 days post-IR. **D.** Bar plot showing the percentage of each cell type in each of the 3 treatment groups**. E.** *Bpifa2* is relatively enriched in de-differentiated (Dd) acini in the IR+S group, as compared to the non-IR and IR+P group. **F.** Violin plots showing gene expression signature in the 4 acinar sub-clusters across the 3 treatment groups. Ac, active. Dd, de-differentiated. RS, resting. Pr, proliferating. **G.** Immunostaining and confocal imaging of acini in cell cycle arrest, as marked by p21. Scale bar, 20µm. Arrows point to $p21^{+}AQP5^{+}$ acini.

bioRxiv preprint doi: https://doi.org/10.1101/2024.12.31.630834; this version posted January 2, 2025. The copyright holder for this preprint (which was not certified by peer review) is the author/funder, who has granted bioRxiv a license to display the preprint in perpetuity. It is made available under aCC-BY 4.0 International license.

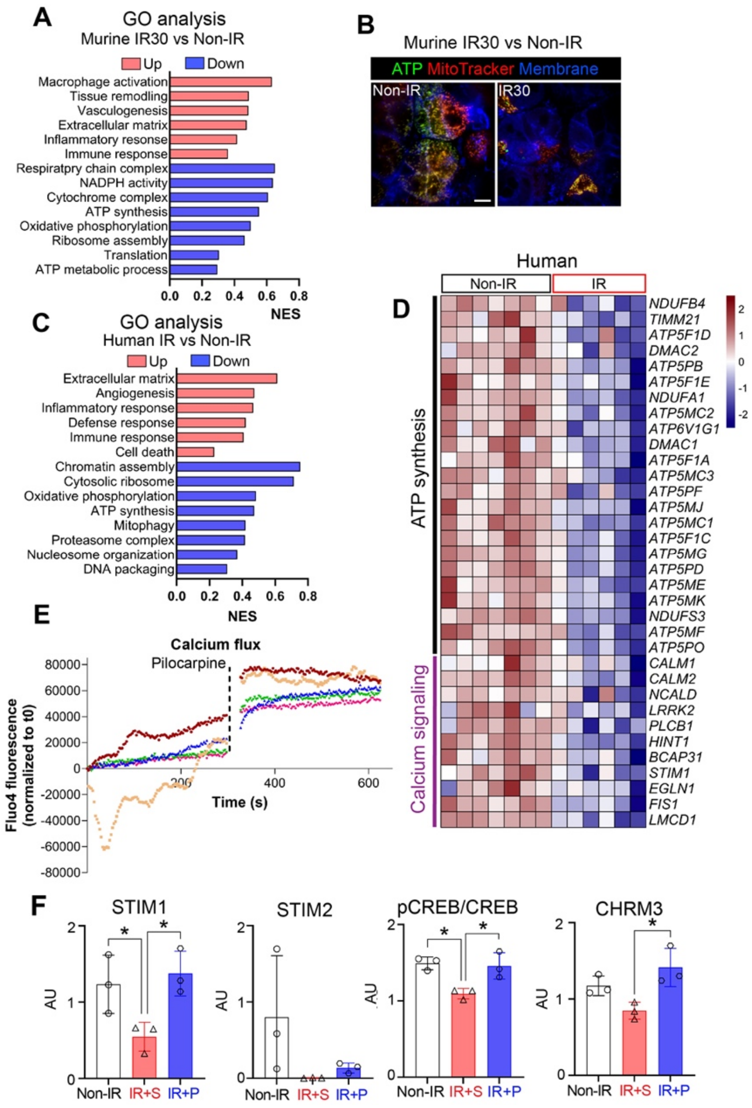

Figure S5. Chronic degeneration is reversed through the restoration of mitochondrial metabolism and calcium signaling, related to Figure 5. A. Gene Ontology (GO) analyses of murine non-IR versus 30 days post-IR SGs bulk RNA-seq datasets. **B.** Live imaging of ATP synthesis and active mitochondria (MitoTracker) in non-IR and 30 days post-IR murine SGs, cultured ex vivo for 24 hours. **C.** GO analysis of human non-IR versus IR SGs (>2 yrs post-radiation, GSE206878[26]) bulk RNA-seq datasets. **D.** Heatmap of ATP synthase and calcium signaling-related gene expression levels in human salivary glands, irradiated (IR) versus healthy (Non-IR). **E.** Calcium signaling levels, with or without muscarinic stimulation, measured by live imaging of Fluo-4 AM uptake by freshly harvested SGs. Each color represents a biological replicate **F.** Quantification of STIM1, STIM2, pCREB and CHRM3 protein levels in each condition determined by western blot (Figure 5F). Groups were normalized to actin. Mean±SD. *, P<0.05.

Table S1: List of antibodies used.

Antibody	Raised in	Dilution	Use	Source	Catalog #
**E-cadherin**	Rat	1:400	IF	Life Technologies	13-1900
**Aquaporin 5**	Rabbit	1:200	IF	Millipore	AB3559
**MUC10**	Goat	1:200	IF	Abcore	AC21-2394
**AChE**	Mouse	1:200	IF	Thermofisher	MA3-042
**Parotid Secretory Protein (PSP)**	Guinea Pig	1:500	IF	Gift from Stefan Ruhl	N/A
**CD3**	Rabbit	1:200	IF	Abcam	Ab5690
**GFRa2**	Goat	1:100	IF	R&D Systems	AF429
**CD31**	Rat	1:300	IF	R&D Systems	AF3628
**SMA**	Mouse	1:300	IF	Sigma-Aldrich	C6198
**DCT**	Rabbit	1:200	IF	abcam	ab221144
**TH**	Rabbit	1:200	IF	Sigma-Aldrich	AB152
**γH2AX**	Rabbit	1:500	IF	Cell Signaling Technology	9718S
**p21**	Rat	1:200	IF	abcam	ab107099
**TUBB3**	Mouse	1:200	IF	Biolegend	801202
**ATP5A1**	Rabbit	1:200	IF	ProteinTech	14676-1-AP
**NKCC1**	Goat	1:400	IF	Santa Cruz	sc-21545
**STIM1**	Rabbit	1:1000	WB	ProteinTech	11565-1-AP
**STIM2**	Mouse	1:1000	WB	Millipore	ABF218
**Phospho-CREB (Ser133)**	Rabbit	1:1000	WB	Cell Signaling Technology	9198S
**CREB1**	Rabbit	1:1000	WB	Millipore	Ab3006
